# ADP-Ribosylation Factor-Interacting Protein 2 Acts as a Novel Regulator of Mitophagy and Autophagy in Podocytes in Diabetic Nephropathy

**DOI:** 10.3390/antiox13010081

**Published:** 2024-01-08

**Authors:** Haihua Guo, Manuel Rogg, Julia Keller, Ann-Kathrin Scherzinger, Julia Jäckel, Charlotte Meyer, Alena Sammarco, Martin Helmstädter, Oliver Gorka, Olaf Groß, Christoph Schell, Wibke Bechtel-Walz

**Affiliations:** 1 Department of Medicine IV, University Medical Center, Faculty of Medicine, University of Freiburg, 79106 Freiburg, Germany; 2 Institute of Surgical Pathology, Medical Center, Faculty of Medicine, University of Freiburg, 79106 Freiburg, Germany; 3 Faculty of Biology, University of Freiburg, 79106 Freiburg, Germany; 4 EMcore, Renal Division, University Medical Center, Faculty of Medicine, University of Freiburg, 79106 Freiburg, Germany; 5 Institute of Neuropathology, Experimental Neuropathology, University Medical Center, Faculty of Medicine, University of Freiburg, 79106 Freiburg, Germany; 6 Freiburg Institute for Advanced Studies (FRIAS), University of Freiburg, 79106 Freiburg, Germany; 7 Berta-Ottenstein Program, Faculty of Medicine, University of Freiburg, 79106 Freiburg, Germany

**Keywords:** ADP-ribosylation factor-interacting protein 2, Arfip2, podocyte, mitophagy, autophagy, diabetes, CKD

## Abstract

(1) Background: Differentiated podocytes are particularly vulnerable to oxidative stress and cellular waste products. The disease-related loss of postmitotic podocytes is a direct indicator of renal disease progression and aging. Podocytes use highly specific regulated networks of autophagy and endocytosis that counteract the increasing number of damaged protein aggregates and help maintain cellular homeostasis. Here, we demonstrate that ARFIP2 is a regulator of autophagy and mitophagy in podocytes both in vitro and in vivo. (2) Methods: In a recent molecular regulatory network analysis of mouse glomeruli, we identified ADP-ribosylation factor-interacting protein 2 (Arfip2), a cytoskeletal regulator and cofactor of ATG9-mediated autophagosome formation, to be differentially expressed with age. We generated an *Arfip2*-deficient immortalized podocyte cell line using the CRISPR/Cas technique to investigate the significance of Arfip2 for renal homeostasis in vitro. For the in vivo analyses of *Arfip2* deficiency, we used a mouse model of Streptozotozin-induced type I diabetes and investigated physiological data and (patho)histological (ultra)structural modifications. (3) Results: *ARFIP2* deficiency in immortalized human podocytes impedes autophagy. Beyond this, *ARFIP2* deficiency in human podocytes interferes with ATG9A trafficking and the PINK1-Parkin pathway, leading to the compromised fission of mitochondria and short-term increase in mitochondrial respiration and induction of mitophagy. In diabetic mice, *Arfip2* deficiency deteriorates autophagy and leads to foot process effacement, histopathological changes, and early albuminuria. (4) Conclusions: In summary, we show that ARFIP2 is a novel regulator of autophagy and mitochondrial homeostasis in podocytes by facilitating ATG9A trafficking during PINK1/Parkin-regulated mitophagy.

## 1. Introduction

Podocytes are highly specialized postmitotic epithelial cells of the renal glomerulus forming the slit diaphragm [1] of the kidney. These multifaceted cells have a very restricted capability to regenerate [1,2]**,** and decreasing numbers predict kidney aging and the progression of renal disease [3,4]. Podocyte injury is the main pathologic process observed in many glomerular diseases, such as minimal change disease, membranous glomerulopathy, focal segmental glomerulosclerosis (FSGS), and diabetic nephropathy (DN). Podocytes maintain cellular homeostasis via sophisticated intracellular pathways; however, excessive stress affects the integrity and function of podocytes. Physiological and pathological stimuli (i.e., mechanical stress, oxidative stress, and immunologic stress) contribute to the disruption of the homeostasis of the glomerular filtration barrier [5]. Podocyte injury represents a crucial factor in the development of albuminuria in DN. In DN, the number of podocyte-specific markers and podocyte numbers themselves are decreased, consequently leading to albuminuria and the further development of glomerulosclerosis [6]. Autophagy is the dynamic multistep cellular process wherein portions of cytoplasm, including organelles, are sequestered into double-membrane vesicles (autophagosomes) and delivered to lysosomes where they are degraded, with the eventual recycling of the resultant macromolecules [7]. By removing excessive and aberrant organelles and proteins, autophagy contributes to cellular homeostasis and protein quality control and functions as a source of energy for the cell [8]. Autophagy is increased and protective when facing cellular stressors, i.e., starvation [9] and ischemia [10]. The kidney represents a prime target of age-associated organ damage. Dysregulated autophagy can cause or worsen acute kidney injury (AKI), DN, polycystic kidney disease, and renal aging [11,12].

Damaged mitochondria are detrimental to cellular homeostasis. The clearance of damaged mitochondria through autophagy is called mitophagy. Current studies on the mechanisms of mitophagy focus on ubiquitin (Ub)-dependent pathways and Ub-independent pathways. The phosphatase and tensin homolog (PTEN) induce the putative kinase 1 (PINK1)/Parkin pathway, which poly-ubiquitylates damaged mitochondria to promote mitophagy and is probably the most widely studied ubiquitin-dependent pathway in the elimination of damaged mitochondria in mammals [13]. In Ub-independent pathways, the mitochondrial outer membrane protein B-cell lymphoma 2 (Bcl-2)-interacting protein 3 (BNIP3)L/Nix and FUN14 domain-containing protein 1’s (Fund1) receptor serve as a mitophagy receptor by directly binding to LC3 without ubiquitination to induce mitophagy [14]. ADP-ribosylation factor-interacting protein 2 (Arfip2) is a Ras-related C3 botulinum toxin substrate 1 (Rac1)-interacting protein and one of the Bin/Amphiphysin/Rvs (BAR) domain proteins, which play a key role in intracellular transport through the trans-Golgi network (TGN) via phosphoinositide PI(4)P binding [15]. Like other BAR domain-containing proteins, Arfip2 can sense membrane curvature depending on ADP-ribosylation factor 1 (Arf1). Its overexpression causes the formation of tubular structures to emanate from the Golgi complex [16]. In humans, ARFIP2 is a substrate of protein kinase B (PKB, also known as Akt) and regulates the aggregated formation of mutant huntingtin in Huntington’s disease, depending on its phosphorylation state [17]. It was recently shown that ARFIP2 can control autophagy through its interaction with autophagy-related 9 (ATG9) in human embryonic kidney cells by regulating the starvation-induced distribution of ATG9-positive vesicles that deliver PI4-kinase PI4KIIIβ to the autophagosome initiation site [18]. A high ARFIP2 expression correlates with the early recurrence and metastasis in hepatocellular carcinoma (HCC) patients, while a low expression of ARFIP2 inhibits HCC cell invasion and migration. Moreover, ARFIP2 silencing blocks HCC cell proliferation, invasion, and migration, whereas its overexpression induces epithelial-to-mesenchymal transition (EMT) and inhibits apoptosis and autophagy via the Akt signaling pathway [19]. In our study, we confirm ARFIP2 as a regulator of the cytoskeleton and co-factor of ATG9-mediated autophagosome formation in podocytes, showing for the first time that ARFIP2 acts as a novel regulator of mitophagic homeostasis in podocytes in diabetic nephropathy by adjusting the PINK1-Parkin pathway and participating in oxidative phosphorylation in vivo and in vitro.

## 2. Materials and Methods

### 2.1. Animals

Mice were housed in a specific pathogen–free facility and kept in a 12 h day/night cycle with free access to chow and water according to the German law for the welfare of animals and to the National Institutes of Health Guide for the Care and Use of Laboratory Animals. The genotyping and breeding of animals was performed according to standard procedures. The Cre-lox and FLP-FRT system was used to create constitutive *Arfip2* knockout mice. Briefly, we purchased C57BL/6N-Arfip2^tm1e(EUCOMM)Wtsi^/Ieg carrying a L1L2_Bact_P cassette at position 77267363 of Chromosome 5 upstream of the critical exons 4, 5 and B6.129S4-Gt(ROSA)26Sor^tm2(FLP*)Sor^/J from the European Mouse Mutant Archive. The L1L2_Bact_P cassette is composed of an FRT site followed by a lacZ sequence and a loxP site. The first loxP site is followed by a neomycin resistance gene under the control of the human beta-*ACTIN* promoter, SV40 polyA, a second FRT site, and a second loxP site. A third loxP site is inserted downstream of the targeted exon(s) at position 77269113. The critical exons 4 and 5 of *Arfip2* are, thus, flanked by loxP sites. We created a conditional ready (floxed) allele by crossing these mice to mice expressing the flp recombinase (Supplemental Figure S1). Subsequent crossings of the resulting mice with STOCK *Edil3^Tg(Sox2-cre)1Amc^*/J mice (a gift from Prof. S. Arnold of the Pharmacology Department of the University of Freiburg) resulted in a constitutive *Arfip2* knockout mouse. Briefly, these Sox2Cre transgenic mice express Cre recombinase under the control of the mouse Sox2 (SRY-box containing gene 2) promoter and may be useful for generating epiblast-derived specific conditional mutations. Sox2 is one of the key factors in the production of inducible stem cells (iPCSs) and functions as a transcription factor that is essential for maintaining self-renewal or the pluripotency of undifferentiated embryonic stem cells [20]. Under the control of the *Sox2*-promotor, cre is expressed early during development, leading to the deletion of the floxed region in all cells. For diabetes experiments, eight-week-old male mice were rendered diabetic through intraperitoneal injections of STZ (S-0130, Sigma-Aldrich, St. Louis, Missouri, USA) (middle-dose-STZ-protocol: 125 µg/g in 50 mM sodium citrate buffer, sterile filtered, adjusted to pH 4.5) on day 1 and day 4. The control mice received a citrate buffer only. Mice with fasting glycemia above 13.9 mmol/L were considered diabetic. Mice were sacrificed 16 weeks after the induction of diabetes. The organs were perfused via the aorta with Paraformaldehyd (PFA) at 4% immediately after harvesting to rinse the blood from the capillaries and to pre-fix the tissue. All animal experiments were approved by the local authorities (Regierungspräsidium Freiburg, G16/85, G16/148, and G21-87).

### 2.2. Genotyping for Mice

A small piece of the tail tip was cut into thin-walled PCR tubes, frozen at −20 °C for at least 30 min (min), and then lysed in 75 μL of an alkaline lysis reagent (pH 12) for 30 min at 95 °C in a PCR cycler. After the samples were cooled to at least 12 °C in a cycler, the reaction was stopped via the addition of 75 μL of the neutralization reagent (pH 5). The eluted DNA could be stored at −20 °C until usage. All genotypings were set up using 2.5 μL templates. The DNA fragments were separated by size in 2% agarose gel and visualized via ethidium bromide (EtBr) and ultraviolet light (UV). The primers used for genotyping are listed in Table 1.

### 2.3. Measurement of Urinary Albumin and Creatinine

Proteinuria, albumin, and creatinine levels were quantified by measuring urine from wildtype and knockout mice collected at defined time points in the respective experiments. Calculating the ACR indicates the extent of proteinuria. The quantification of urinary albumin was performed using the Albumin Blue Fluorescent Assay Kit (Active Motif, #15002, Carlsbad, CA, USA) and Mouse Albumin Enzyme-linked Immunosorbent Assay (ELISA) (Abcam, ab108792). Urinary creatinine was measured using the creatinine PAP LT-SYS kit (Labor & Technik, LT-CR 0106).

### 2.4. Antibodies

The antibodies and dilutions used in this study are listed in Table 2.

### 2.5. Histologic Analyses

The periodic acid-Schiff (PAS) staining of formaldehyde-fixed paraffin-embedded tissue (FFPE) kidney sections (2 µm) was performed applying standardized diagnostic procedures at the Institute of Surgical Pathology (University of Freiburg) as previously described [21]. The digitalization of slides was realized using a Ventana DP 200 slide scanner (Roche Diagnostics Deutschland GmbH, Mannheim, Germany). The quantification of glomerular sclerosis (GS) of >50 glomeruli per animal was performed as previously described [21]. In brief, glomeruli were graded by applying a 5-tier score (0—no sclerosis, 1—mesangial expansion or glomerular capillary thickening or adhesion of the glomerular tuft and Bowman’s capsule, 2—<50% sclerosis of the glomerular tuft, 3—>50% sclerosis, 4—obliteration of the glomerular tuft). The mean GS score per animal was calculated and used for statistical analysis.

### 2.6. Electron Microscopy

For ultrastructural analysis using Transmission electron microscopy (TEM), kidneys and podocyte cell cultures were fixed in 4% PFA and 2% glutaraldehyde (#4157, Carl Roth, Karlsruhe, Germany) in a 0.1 M cacodylate buffer (#11650, Science Services, München, Germany). The samples were postfixed in 1% osmium tetroxide (Science Services, #E19150) in double-distilled water (ddH_2_O) and then washed six times in ddH_2_O. The tissue was stained en bloc in 1% uranyl acetate solution (Science Services, #E22400-1) and washed two times in ddH_2_O. Dehydration was performed via sequential incubation steps in 30%, 50%, 70%, 90%, and 2 × 100% ethanol (#32205, Fisher Scientific, Hampton, NH, USA) and then 2× 100% acetone (#179124, Sigma-Aldrich, St. Louis, MO, USA). All incubation steps were microwave-assisted (BioWave Pro+, PELCO, Fresno, CA, USA). After embedding in Durcupan resin (Sigma-Aldrich, #44611 and #44612), ultrathin sections (55 nm) were performed using a UC7 ultramicrotome (Leica), collected on Formvar-coated (Science Services, #E15830-25) copper grids (#G2500C, Plano GmbH, Wetzler, Germany). Post-staining was conducted for 1 min with 3% lead citrate (#11300, Delta Microscopies, Mauressac, France), and imaging was performed using a Talos L120c TEM (ThermoFisher, Waltham, MA, USA).

### 2.7. Electron Microscopy Image Analyses

Random electron microscopy images were used for the morphometric analysis of foot process effacement. Three glomeruli of *n =* 2 mice each were evaluated. Electron images were analyzed using ImageJ software. The quantification of foot process effacement was adapted from van den Berg et al. [22]. Briefly, from each picture, the mean width of the foot processes (FPWs) was calculated according to the following formula: FPW = π/4 × (Σ glomerular basement membrane length/Σ foot process). A foot process was defined as any connected epithelial segment butting on the basement membrane between two neighboring filtration slits. Data are the means ± standard error of the mean (SEM); * *p* < 0.05. Mitochondria of podocyte cell culture TEM images were quantified using ImageJ software, analyzing twenty mitochondria per cell in ten *ARFIP2*-deficient podocytes and control podocytes, respectively. The quantification of the area of mitochondria was adapted from Lam et al. [23].

### 2.8. In Situ Hybridization and LacZ Staining

The in situ hybridization and LacZ staining of kidney sections were previously described in detail [24]. The primers used for the generation of in situ probes are described in Table 1. Overview images of in situ hybridizations are stitched from multiple pictures.

### 2.9. Immunofluorescence Stainings

After PFA fixation and dehydration in a histocinette, the kidneys were embedded in paraffin. Then, the kidneys were sliced into 3 μm thin sections, placed on glass slides, and dried at 42 °C overnight. The slices were dewaxed in Histo-Clear^®^ for 2 × 10 min and rehydrated using 100% ethanol for 2 × 5 min and in 90%, 80%, 70%, 50%, 30% ethanol and 1× phosphate-buffered saline (PBS) for 2 min. Heat-induced epitope retrieval was then performed in tris(hydroxymethyl)aminomethane (TRIS)/ethylenediaminetetraacetic acid (EDTA) pH9 for 30 min using a food steamer with the highest pressure. After cooling down on the ice for 30 min and washing in 1× PBS for 3 × 3 min, the slices were encircled using a super peroxidase-antiperoxidase (PAP) pen liquid blocker and incubated in a humidified chamber at room temperature for about 2 h in 5% Bovine serum albumin (BSA) in 1× PBS to reduce an unspecific background signal. The blocking solution was then replaced by the primary antibodies diluted in 5% BSA in 1× PBS. After 45 min, the slides were washed in 1× PBS for 3 × 3 min to remove the unbound antibodies. Together with blue fluorescent dye to stain DNA (Hoechst stain), the appropriate secondary antibodies were diluted in 5% BSA in 1× PBS and incubated on the slices for 30 min. Hence, the slides were washed again in 1× PBS for 3 × 3 min, covered carefully with Prolong Gold Antifade and glass cover, and sealed with clear nail polish the next day. Pictures were taken using a ZEISS LSM 980 Confocal Microscope with 10×, 20×, and 63× objectives.

### 2.10. Cell Culture

Human-immortalized podocytes (AB8/13) were kindly provided by Moin A. Saleem (Bristol Medical School, University of Bristol, Bristol, UK) and transduced to stably express green fluorescent protein (GFP)-microtubule-associated proteins of the 1A/1B light chain 3B (LC3) via a lentiviral expression vector (kind gift of Noboru Mizushima, Department of Biochemistry and Molecular Biology, University of Tokyo, Japan). Podocytes were cultured in Roswell Park Memorial Institute (RPMI) 1640 (ThermoFisher Scientific, #61870036, Waltham, MA, USA) supplemented with a 10% fetal bovine serum (FBS), 10 µg/mL of insulin/transferrin/selenite (Sigma-Aldrich, #11074547001), 1% penicillin/streptomycin (ThermoFisher Scientific, #15140122), 100 µM of sodium-pyruvate (ThermoFisher Scientific, #11360-039), 1 µM of a non-essential amino acids solution (ThermoFisher Scientific, #11140035) and 5 mM of a HEPES (4-[2-hydroxyethyl]-1-piperazineethanesulfonic acid) buffer solution (ThermoFisher Scientific, #15630-056) at 33 °C in 95% O_2_ and 5% CO_2_ according to standard cell-culture procedures to keep an undifferentiated state. When the cells had grown to 60% confluence, differentiation was induced by changing the temperature to 37 °C with 5% CO_2_ for 10-14 days.

### 2.11. CRISPR/Cas9 and Lentiviral Transduction

*ARFIP2*-knockout (KO) cells were generated using CRISPR/Cas9 genome editing technology in immortalized human podocytes. Single-guide RNAs (gRNAs) targeting the human *ARFIP2* gene were designed using the website-based platform CHOPCHOP (https://chopchop.cbu.uib.no, accessed on 15 September 2020). gRNA (5′-GAGCGTGTCTTCCATGGTCTTGG-3′) was subcloned into the TLCV2 plasmid (Addgene plasmid #87360; https://www.addgene.org/87360/, accessed on 15 September 2020). TLCV2 plasmids (without gRNA) were used to generate negative (wildtype) control cells. Lentiviral particles were produced in HEK 293T cells according to the standard protocols. Immortalized human podocytes were transduced using lentiviral particles, while single-cell clones of CRISPR/Cas9 genome-edited podocytes were generated. *ARFIP2*-KO in single-cell clones of podocytes was validated using Western blot analysis.

### 2.12. Western Blot Analysis

Western Blot was performed using cell lysates or lysates from kidney cortexes. Cell lysates were generated via lysing the cell pellet in a Radio Immuno Precipitation Assay (RIPA) lysis buffer for 15 min on ice. After centrifugation at 14,000× rpm for another 15 min, the supernatants were denatured in a 2× Lämmli buffer supplemented with dithiothreitol (DTT) at 95 °C for 5 min. Tissue lysates were generated via homogenization in a glass pestle in the RIPA buffer, followed by denaturation in 2× Lämmli with DTT after centrifugation. Measurements of the whole protein amount were performed using the Pierce bicinchoninic acid (BCA) Protein Assay Kit (ThermoFisher Scientific, #23225), according the manufacturer’s instruction. Equal amounts of protein were loaded for standard SDS-polyacrylamide gel electrophoresis (SDS-PAGE) and Western Blot was performed using the trans-blot turbo transfer system (Bio-Rad Laboratories, Inc., Hercules, CA, USA) including polyvinylidene difluoride (PVDF) membranes (Bio-Rad, #1704157, #1704156). After blocking the membranes for 1 h with 5% BSA in 1× Tris-Buffered Saline and 0.1% Tween^®^ 20 Detergent (TBST), the membranes were incubated with primary antibodies in 5% BSA in TBST for 24 h at 4 °C. Afterwards, the membranes were washed with TBST and then incubated with Horseradish peroxidase (HRP)-linked secondary antibodies in TBST for 1 h at room temperature. After washing, the blots were imaged with HRP-linked chemiluminescence (self-made ECL/Biozym, #541014/ThermoFisher Scientific, #34580) using either X-ray films in a Curix 60 developer machine from Aktien-Gesellschaft für Anilin-Fabrikation (AGFA) or the Advanced Fluorescence Imager from Intas without X-ray films.

### 2.13. Analysis of Cellular Reactive Oxygen Species (ROS) Levels

Intracellular ROS levels were detected using the dichlorodihydrofluorescein diacetate (DCFDA)/2′,7′-Dichlorodihydrofluorescein-diacetat (H_2_DCFDA)-cellular ROS assay kit (ab113851, Abcam, United States). Briefly, the immortalized human podocytes were seeded in 96-well plates (20 × 10^3^ cells/well) to allow cells to adhere overnight at 33 °C in 95% O_2_ and 5% CO_2_. The next day, cells were washed with a 1× buffer and then incubated with a diluted DCFDA solution (50 µmol) at 37 °C for 45 min in the dark. Next, the cells were twice washed with the 1× buffer. Finally, ROS expression was observed and measured on a plate reader (from TECAN) at Ex/Em = 485/535 nm. In our study, tert-butyl hydroperoxide (TBHP) (50 µmol) was used as a positive control, which confirmed that cell lines produced ROS.

### 2.14. Analysis of Mitochondria Stress and Glycolytic Activity In Vitro

Analyses of mitochondria stress and glycolytic activity were performed using a Seahorse XFp96 Analyzer according to the manufacturer’s instructions (Agilent Technologies). Briefly, the optimization of the cell density of immortalized human podocytes, as well as the optimization of the working concentration titers for each individual inhibitor, was conducted prior to the analyses. Cells were seeded at a density of 20,000 cells/well. The Mito Stress Test and glycolysis stress were performed following the manufacturer`s instructions. Specifically, podocytes on XFp microplates were rinsed, and an XF assay buffer was added. Afterward, the plate was equilibrated for 1 h at 37 °C in a non-CO_2_ incubator. All mediums and solutions of mitochondrial complex inhibitors were adjusted to pH 7.4 on the day of the assay. Following four baseline measurements of the oxygen consumption rate (OCR) and extracellular acidification rate (ECAR), inhibitors of the respiratory chain were sequentially injected into each well. Three OCR and ECAR readings were taken after the addition of each inhibitor before the automated injection of the subsequent inhibitor. Mitochondrial complex inhibitors included oligomycin (1 μM) to inhibit complex V, carbonyl cyanide-p-trifluoromethoxyphenylhydrazone (FCCP) (0.5 μM) to uncouple the proton gradient, antimycin A (0.5 μM, inhibitor of complex III), and rotenone (0.5 μM, complex I inhibitor). The glycolytic activity compound includes glucose (10 mM), oligomycin (1 μM), and 2-deoxy-D-glucose (2-DG) (50 mM). OCR and ECAR were automatically calculated using Seahorse XFp software version 2.2.0 (Seahorse Bioscience, Billerica, MA, USA).

### 2.15. Image Analyses

Image editing was performed using the ZEN 2 blue edition software (Zeiss), Adobe Photoshop CS6, and Adobe Illustrator CS6. All figures and schematics were generated with Adobe Illustrator CS6. Pictures from other sources are marked accordingly. The quantification of fluorescence intensity and area of glomeruli was performed using ImageJ software 1.49 k. The stated value is based on the measured fluorescence per glomeruli, while the displayed mean values of wildtype (WT) and knockout (KO) result from the mean fluorescence per animal. All mouse documentation, including body and kidney weight, the calculation of kidney/body weight, and the quantification and the calculation of albumin creatinine ratio (ACR) were performed with Microsoft Office Excel 2010.

### 2.16. Statistics

Data are expressed as mean values + the standard error of the mean (SEM) if not stated otherwise. Scatter dots indicating individual data points were used for statistical analysis. Unpaired *t*-test, the Mann–Whitney test, or the one-way ANOVA test (Tukey multiple comparison test) were used on the basis of data distribution. GraphPad Prism 9 software was used for analysis. Statistical significance was defined as * *p* < 0.05, ** *p* < 0.01, *** *p* < 0.001, and **** *p* < 0.0001.

## 3. Results

### 3.1. Arfip2 Is Expressed in Podocytes

**Figure 1:**
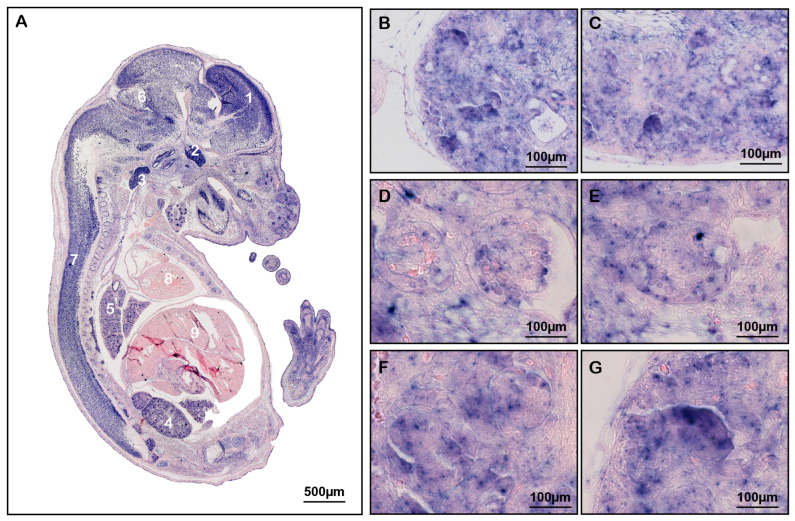
The expression of Arfaptin-2 (Arfip2) in wildtype mice. (**A**): In situ hybridization of E 15.5 embryo. Scale bar 500 μm. 1: forebrain; 2: trigeminal ganglion; 3: vagal ganglion; 4: kidney; 5: lung; 6: mid- and hindbrain; 7: spinal cord; 8: heart; 9: liver. (**B**,**C**): outer region of the kidney; (**D**–**G**): Internal region of the kidney. Scale bars 100 μm.

The general messenger ribonucleic acid (mRNA) expression of *Arfip2* was analyzed via in situ hybridization on sections of E15.5 wildtype embryos to test the distribution of *Arfip2* during murine development (Figure 1). Purple staining indicates the mRNA of *Arfip2*, which is highly enriched in the forebrain, trigeminal ganglion, vagal ganglion, kidney, and lung, present in the mid- and hindbrain, spinal cord, but nearly absent in the heart and liver (Figure 1A). In the kidney, *Arfip2* is expressed in the whole renal tissue, whereas it is mainly enriched in the outer cortex region, the nephrogenic zone (Figure 1B–G).

### 3.2. ARFIP2 Is a Regulator of Autophagy in Podocytes In Vitro

**Figure 2:**
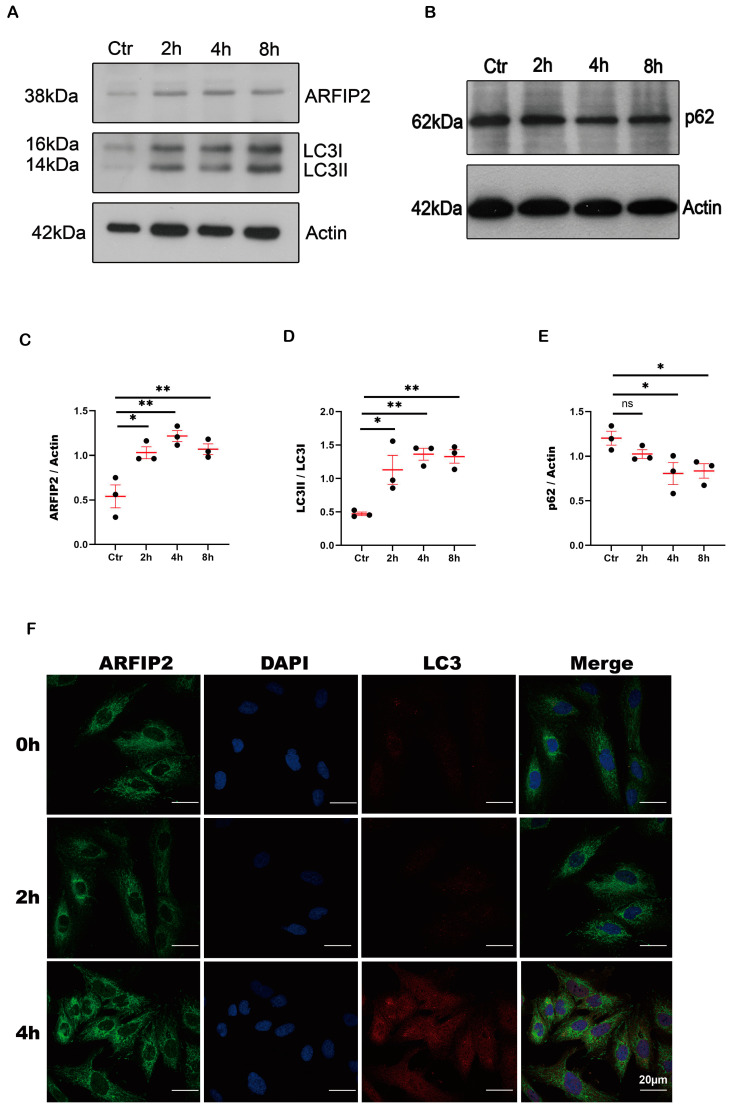
Arfaptin-2 (*ARFIP2)* is a regulator of autophagy in podocytes. Human-immortalized podocytes were incubated with Hanks’ Balanced Salt Solution (HBSS) for 2, 4, or 8 h. The cells were lysed, and the protein was isolated after 2, 4, and 8 h for Western blot analyses. The experiment was carried out three times per condition (*n* = 3). (**A**): Western blot analysis shows increased *ARFIP2* expression and LC3 with increasing starvation time. (**B**): Western blot analysis shows decreased p62 expression with increasing starvation time. (**C**–**E**): The quantification of ARFIP2, LC3 and p62 expression under starvation. One-way ANOVA was used to analyze. (**F**): Immunofluorescence staining shows *ARFIP2* upregulation after starvation for 2 and 4 h. Scale bars 20 µm. * *p* < 0.05, ** *p* < 0.01.

Autophagy can be induced by stress (starvation, hypoxia, endoplasmic reticulum stress, and oxidative stress). Previous research showed that ARFIP2 acts as a transporter molecule of ATG9 to regulate starvation-induced autophagy in human embryonic cells [25]. In our study, we used human-immortalized podocytes to investigate starvation-induced autophagy. The protein levels of ARFIP2 and the microtubule-associated protein 1 light chain 3A (LC3I)’s conversion into the lipid-modified form of LC3 (LC3II) increased significantly while p62 decreased following starvation-induced autophagy after 2, 4, and 8 h of incubation with Hanks’ Balanced Salt Solution (HBSS) (Figure 2A–C).

### 3.3. Autophagy Is Impaired in ARFIP2-Deficient Podocytes

**Figure 3:**
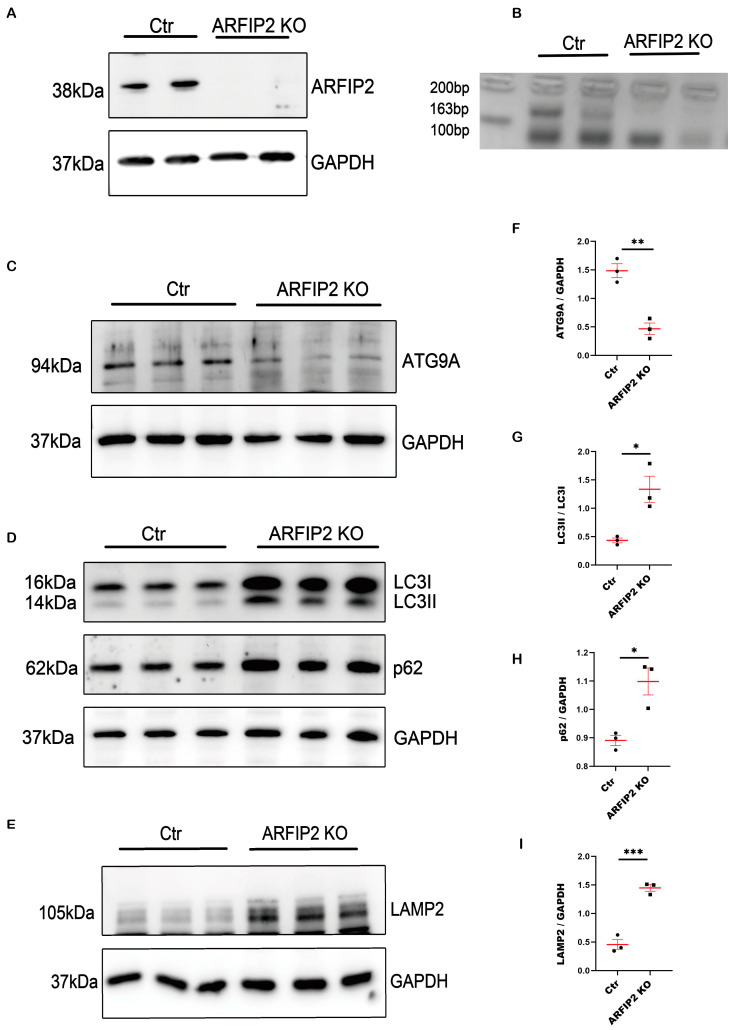
Autophagy is impaired in Arfaptin-2 (*ARFIP2)*-deficient podocytes. (**A**,**B**): Western blot analysis and the PCR-based confirmation of successful CRISPR/cas-based *ARFIP2* knockout in human-immortalized podocytes (one single-cell wildtype clone *ARFIP2* knockout clone was generated, respectively). (**C**,**D**): Western blot analyses showing the changes in ATG9A, microtubule-associated proteins 1A/1B light chain 3B (LC3) and p62 in *ARFIP2*-deficient podocytes (*n =* 3). (**E**): Western blot analysis of the lysosomal marker Lamp2 in *ARFIP2*-deficient podocytes (*n* = 3). (**F**–**I**): Quantification of Western blot analyses displays a significant decrease in autophagy-related protein 9A (ATG9A), a higher LC3II/LC3I ratio with the accumulation of p62 and Lysosome-associated membrane protein 2 (Lamp2). *t*-tests, two-tailed, were used to analyze. Wildtype controls (Ctr); ARFIP2 knockout podocytes (ARFIP2 KO); Glyceraldehyde 3-phosphate dehydrogenase (GAPDH). * *p* < 0.05, ** *p* < 0.01, *** *p* < 0.001.

To further analyze ARFIP2-dependent mechanisms in autophagy, we used CRISPR/Cas9 genome-editing technology to generate the first *ARFIP2*-deficient immortalized human podocyte cell line. We successfully generated monoclonal *ARFIP2* KO clones from one independent single gRNA and confirmed the loss of ARFIP2 via Western blot and PCR analyses (Figure 3A,B). We used one of two selected clones for further analyses; every experiment was carried out three times per condition.

In *ARFIP2*-deficient cells, autophagy-related protein 9A (ATG9A) levels are undetectable (Figure 3C,F). During the induction of autophagy, LC3I is converted into LC3II, which ultimately migrates to the outer membrane of the forming autophagosome, followed by fusion to the latter. Western blot analyses show increased levels of LC3I and LC3II in *ARFIP2*-deficient podocytes. We assume that LC3 conjugation can still occur despite the lack of ARFIP2 (Figure 3D,G). Furthermore, we observed an increase in the Polyubiquitin-Binding Protein P62/SQSTM1 (referred to as p62) in *ARFIP2*-deficient podocytes. p62 is involved in the degradation of misfolded proteins through autophagy, leading to the degradation of p62 when autophagy is induced and vice versa to an accumulation of p62 when autophagy is inhibited. In *ARFIP2*-deficient podocytes, we confirm the accumulation of p62 (Figure 3D,H) and lysosome-associated membrane protein 2 (LAMP2) in *ARFIP2*-deficient podocytes (Figure 3E,I).

### 3.4. ARFIP2 Deficiency in Human Podocytes Impairs Autophagy under Low Glucose Conditions

**Figure 4:**
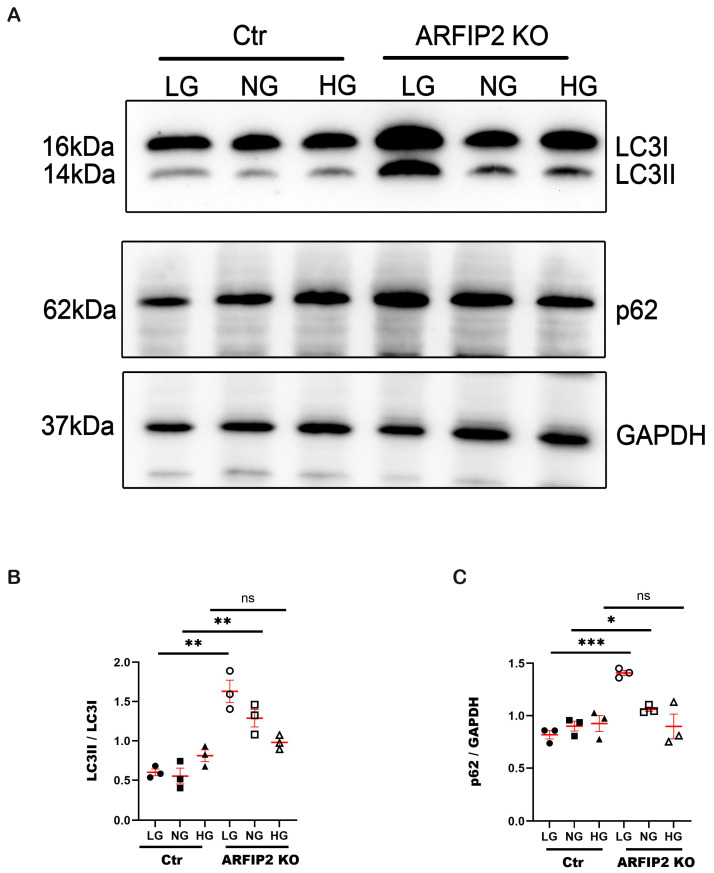
Arfaptin-2 (*ARFIP2)* knockout podocytes show impaired autophagy in low-glucose conditions. (**A**): Western blot analysis shows the changes in the microtubule-associated proteins 1A/1B light chain 3B (LC3II and I) ratio and p62 in *ARFIP2*-deficient podocytes after treatment with low glucose (5 mM), normal glucose (11 mM) or high glucose (30 mM) conditions for 24 h (*n =* 3). (**B**,**C**): The quantification of Western blot analyses displays significantly higher LC3II/LC3I and the accumulation of p62 when incubated with low glucose and normal glucose. *t*-tests, two-tailed, are used to analyze each group. Wildtype controls (Ctr); ARFIP2 knockout podocytes (ARFIP2 KO); Glyceraldehyde 3-phosphate dehydrogenase (GAPDH). * *p* < 0.05, ** *p* < 0.01, *** *p* < 0.001.

Autophagy can be induced differently via glucose starvation in *ARFIP2*-deficient and control podocytes. We incubated cells in low glucose medium (LG) (5 mmol), normal glucose medium (NG) (11 mmol), and high glucose medium (HG) (30 mmol) and observed a significant increase in LC3II accompanied by the accumulation of p62 in *ARFIP2*-deficient podocytes in low glucose conditions (Figure 4A–C), indicating how *ARFIP2* deficiency inhibits autophagy.

### 3.5. Autophagy Flux Is Disrupted in ARFIP2 Deficient Podocytes

**Figure 5:**
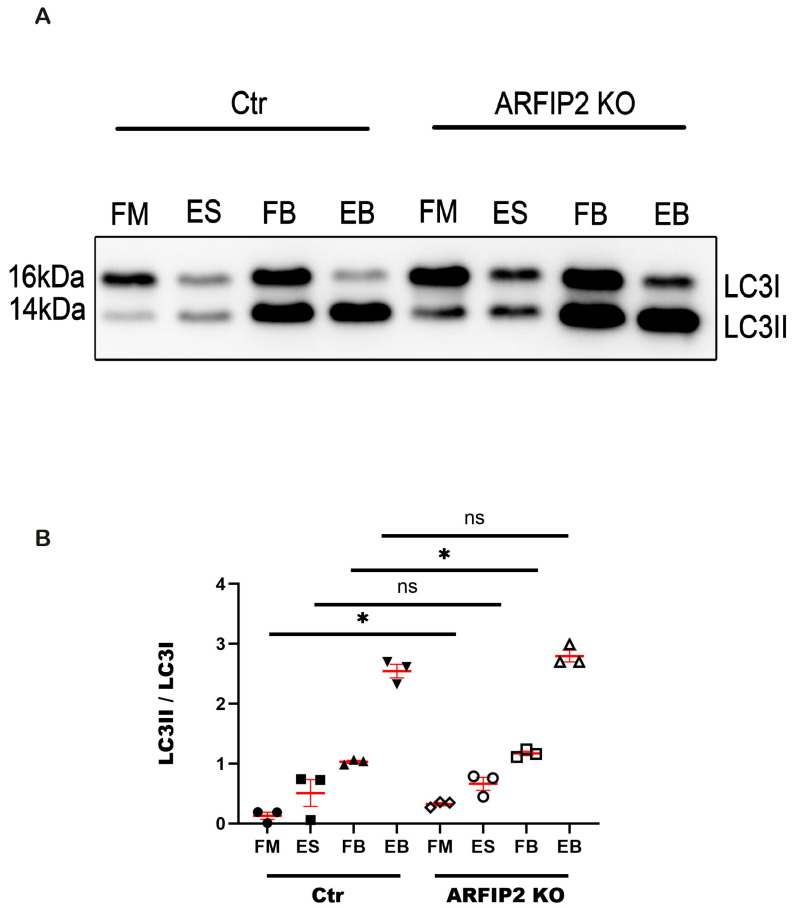
Autophagy flux is partially blocked in Arfaptin-2 (*ARFIP2)*-deficient podocytes. (**A**): *ARFIP2*-knockout podocytes and controls were starved in the presence or absence of 100 μM of BafA1 (*n =* 3). (**B**): The quantification of Western blot analyses displays increased microtubule-associated proteins 1A/1B light chain 3B II and I (LC3 II and I) ratio under normal conditions and with BafA1. *t*-tests, two-tailed, were used for analyses. Wildtype controls (Ctr); ARFIP2 knockout podocytes (ARFIP2 KO); Glyceraldehyde 3-phosphate dehydrogenase (GAPDH). FM: full medium. ES: starvation condition. FB: full medium with BafA1. EB: starvation with BafA1. * *p* < 0.05.

As a characteristic sign of blocked autophagy, LC3 and p62 accumulate into *ARFIP2*-deficient podocytes. To investigate autophagic flux, wildtype controls, and *ARFIP2*-deficient podocytes were starved in the presence or absence of 100 μM of Bafilomycin A1 (BafA1). BafA1 is a specific and reversible inhibitor of vacuolar H^+^-ATPase, which blocks the autophagosome–lysosome fusion. We observed a higher LC3II/LC3I ratio under normal conditions in ARFIP2-deficient podocytes without starvation. After 4 h of starvation plus the blocking of the autophagy flux, we observed a small increase in LC3I into LC3II after BafA1 treatment in *ARFIP2*-deficient podocytes, though there was no difference compared with the controls; autophagic flux was not inhibited (Figure 5).

### 3.6. The PINK1-Parkin Pathway, Mitochondrial Fission and Mitophagy Are Affected in ARFIP2 Deficient Podocytes

**Figure 6:**
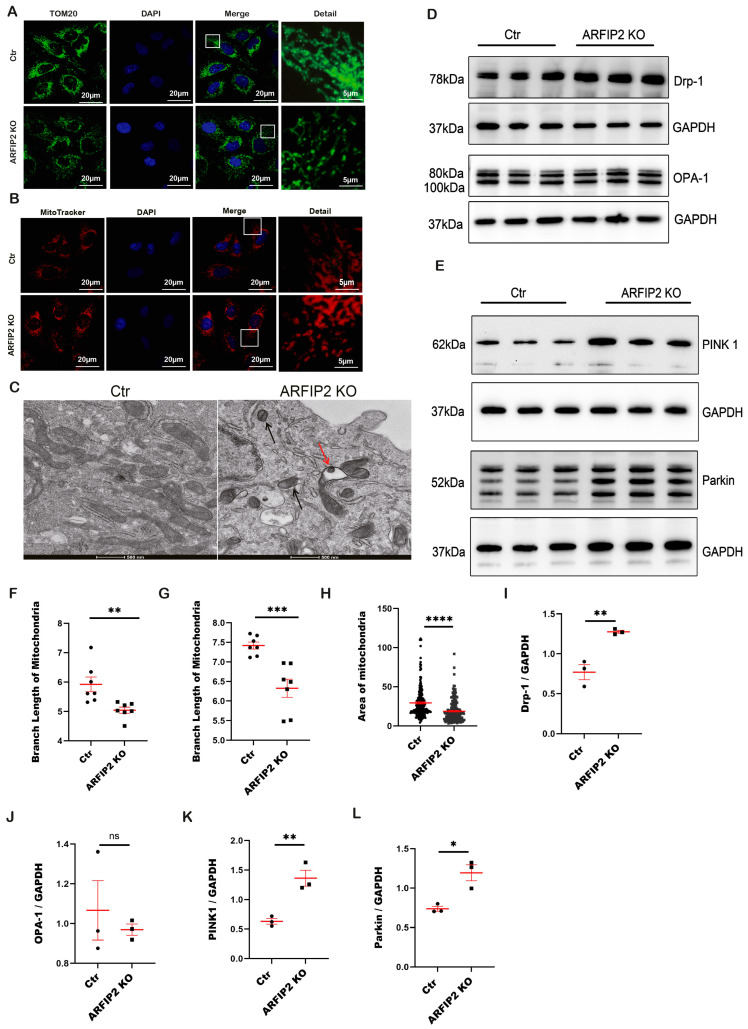
Impaired fission of mitochondria and the PINK1-Parkin pathway regulate the induction of mitophagy in Arfaptin-2 (*ARFIP2)*-deficient podocytes. (**A**,**B**): Confocal microscopy analyses of immunofluorescence staining of the mitochondrial import receptor subunit TOM20 homolog (TOM20) and Mito Tracker™ in human-immortalized *ARFIP2* knockout podocytes and controls. Scale bars 20 µm, detail 5 µm. (**C**): The transmission electron microscopy of *ARFIP2* knockout podocytes and controls (*n =* 10). In total, 20 mitochondria per cell were analyzed. *ARFIP2* knockout podocytes demonstrate decreased numbers of mitochondria. The mitochondria of ARFIP2 knockout podocytes appear rounded. Black arrows show round mitochondria; a red arrow shows a swollen mitochondrium. Scale bar 500 nm. (**D**): Western blot analysis of mitochondrial markers dynamin-1-like protein (Drp-1) and dynamin-like 120 kDa protein, and mitochondrial (OPA-1) in *ARFIP2* knockout podocytes and controls. (**E**): Western blot analysis showing an increased phosphatase and tensin (PTEN)-induced kinase 1 (PINK1)/Parkin pathway in *ARFIP2*-deficient podocytes. (**F**,**G**): The quantification of the branch length of TOM20 and Mito Tracker™, respectively. Seven confocal pictures were used to analyze the total 35 cells per cell line. (**H**): Quantification of mitochondrial area. The area of mitochondria in *ARFIP2* knockout podocytes is significantly smaller than the controls. (**I**–**L**): Quantifications of Western blot analyses display the changes in Drp-1, OPA-1, PINK1 and Parkin in *ARFIP2* knockout podocytes (*n =* 3). *t*-tests, two-tailed, are used to analyze. Wildtype controls (Ctr); ARFIP2 knockout podocytes (ARFIP2 KO); Glyceraldehyde 3-phosphate dehydrogenase (GAPDH). * *p* < 0.05, ** *p* < 0.01, *** *p* < 0.001, **** *p* < 0.0001.

Recently, ARFIP2 has been suggested as an important factor for ATG9A trafficking during PINK1/Parkin mitophagy [26]. When mitochondria are damaged or dysfunctional, they are eliminated via mitophagy, a special form of autophagy. The translocase of the outer membrane (TOM) is a vital mitochondrial transport system facilitating the importation of nuclear-encoded proteins into the organelle. Mitochondrial dysfunction, including the perturbation of the OXPHOS complex, is evident in many diseases, including Alzheimer’s disease. The mitochondrial import receptor subunit TOM20 homolog (TOM20) serves as a marker of the outer membrane of mitochondria. In *ARFIP2*-deficient podocytes, we could observe a smaller branch length with dispersed mitochondria (Figure 6A,F). We used MitoTracker™ to further analyze the morphology of mitochondria. Indeed, we showed that in *ARFIP2*-deficient podocytes, there were also decreased branch lengths of mitochondria compared to the control cells (Figure 6B,G). Electron microscopy revealed distinctly fragmented mitochondria in podocytes (Figure 6C). The mitochondria of *ARFIP2*-deficient podocytes appeared smaller, rounded, and swollen compared to mitochondria in control podocytes (Figure 6C). Overall, the mitochondria numbers were reduced in *ARFIP2* knockout podocytes (Figure 6H). The fission and fusion of mitochondria always keep balance in a cell. Therefore, mitochondrial fragmentation is related to increased mitochondrial fission. Dynamin-related protein-1 (Drp1) is a large GTPase of the dynamin superfamily that plays an important function in mitochondrial fission in mammalian cells. During the process of mitochondrial fission, Drp1 is recruited from the cytosol to the outer mitochondrial membrane. In *ARFIP2*-deficient podocytes, we detected increased DRP1 levels related to high mitochondrial fission, whereas there were no changes in optic atrophy-1 (OPA1), a stress-sensitive mechanism of mitochondrial dynamic homeostasis (Figure 6D,I,J). PINK1 regulates Parkin translocation in impaired mitochondria and drives their removal via mitophagy. We assumed that *ARFIP2* deficiency promotes the fission and elimination of mitochondria through the PINK1-Parkin pathway (Figure 6E,K,L).

### 3.7. ARFIP2 Deficiency Leads to Increased Mitochondrial Respiration

**Figure 7:**
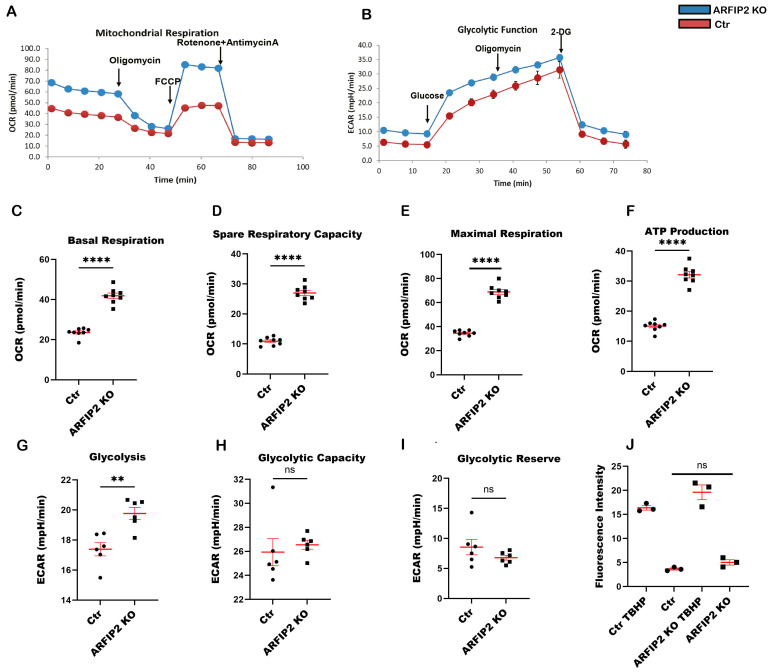
Mitochondrial respiration and glycolytic function are altered in Arfaptin-2 (ARFIP2) knockout podocytes. The oxygen consumption rate (OCR) and extracellular acidification rate (ECAR) were measured using the Seahorse XFp96 Analyzer (Agilent). (**A**): OCR from ARFIP2-deficient podocytes and controls at the baseline and after subsequent injections of oligomycin; Carbonyl cyanide-p-trifluoromethoxyphenylhydrazone (FCCP), and rotenone + antimycin A. Final concentrations: oligomycin: 1 μM; FCCP: 0.5 μM; rotenone: 0.5 μM; antimycin A: 0.5 μM. Basel, spare respiratory capacity, maximal respiration, and ATP production were analyzed in *ARFIP2* knockout podocytes and control podocytes (*n =* 8). (**B**). Glycolytic function (ECAR) was measured using the Seahorse XFp96 Analyzer (Agilent) (*n =* 6). Glycolysis, glycolytic capacity, and glycolytic reserve were analyzed in *ARFIP2* knockout podocytes and respective controls after glucose administration and the subsequent injection of oligomycin and 2-Deoxy-D-glucose (2-DG). Final concentrations: glucose: 10 mM; oligomycin: 1 μM; 2-DG: 50 mM. (**C**–**I**): Quantifications of basal respiration, maximal respiration, ATP production, spare respiratory capacity, basic glycolysis, glycolytic capacity, and glycolytic reserve. The Mann–Whitney test was used for the quantification of Basal respiration. Other quantifications were used as *t*-tests, two-tailed. (**J**): Quantitative 5-(and 6)-Chloromethyl-2′,7′ Dichloro-dihydrofluorescein diacetate (CM-H2DCFDA) fluorescence analyses were performed on *ARFIP2* knockout podocytes and wildtype controls after incubation in 50 µmol of tert-Butyl hydroperoxide (TBHP) to confirm that cell lines produced reactive oxygen species (ROS) (*n =* 3). Cells were incubated in 50 umol of the Dichlorodihydrofluorescein-diacetat (DCFDA) solution for 45 min at 37 °C in the dark. *ARFIP2* knockout podocytes produce more ROS after DCFDA treatment (*p*-value = 0.0549). Wildtype controls (Ctr); ARFIP2 knockout podocytes (ARFIP2 KO); Wildtype control podocytes treated with TBHP (Ctr TBHP); ARFIP2 knockout podocytes treated with TBHP (ARFIP2 KO TBHP). ** *p* < 0.01, **** *p* < 0.0001.

Mitochondria are the primary cellular source of Adenosine triphosphate (ATP). Glycolysis and oxidative phosphorylation (OXPHOS) are the two major pathways to provide energy. To investigate the bioenergetic profile of *ARFIP2*-deficient podocytes and wildtype controls, we measured the oxygen consumption rate (OCR) (Figure 7A) and extracellular acidification rate (ECAR) (Figure 7B) using the Agilent Seahorse XF Flux Analyzer (Agilent). We observed an increased OCR in *ARFIP2*-knockout podocytes and also increased basal respiration, maximal respiration, ATP production, and spare respiratory capacity in *ARFIP2*-deficient podocytes (Figure 7C–F). There was only an increase in basic glycolysis, no difference in glycolytic capacity, and a glycolytic reserve for ECAR (Figure 7G–I). Mitochondria are the main ATP provider, and ARFIP2, as a regulator of mitophagy, apparently participates in the OXPHOS pathway to provide ATP. Since mitochondria are the main source of cellular ROS, we compared ROS generation in wildtype podocytes and *ARFIP2*-deficient podocytes. *ARFIP2*-deficient podocytes generated visibly more ROS, though, in our study, it was not significant (*p*-value 0.0549) (Figure 7J). Wildtype controls (Ctr); ARFIP2 knockout podocytes (ARFIP2 KO).

### 3.8. Arfip2 Deficiency Deteriorates Kidney Function in STZ Induced Type I Diabetic Nephropathy in Mice

**Figure 8:**
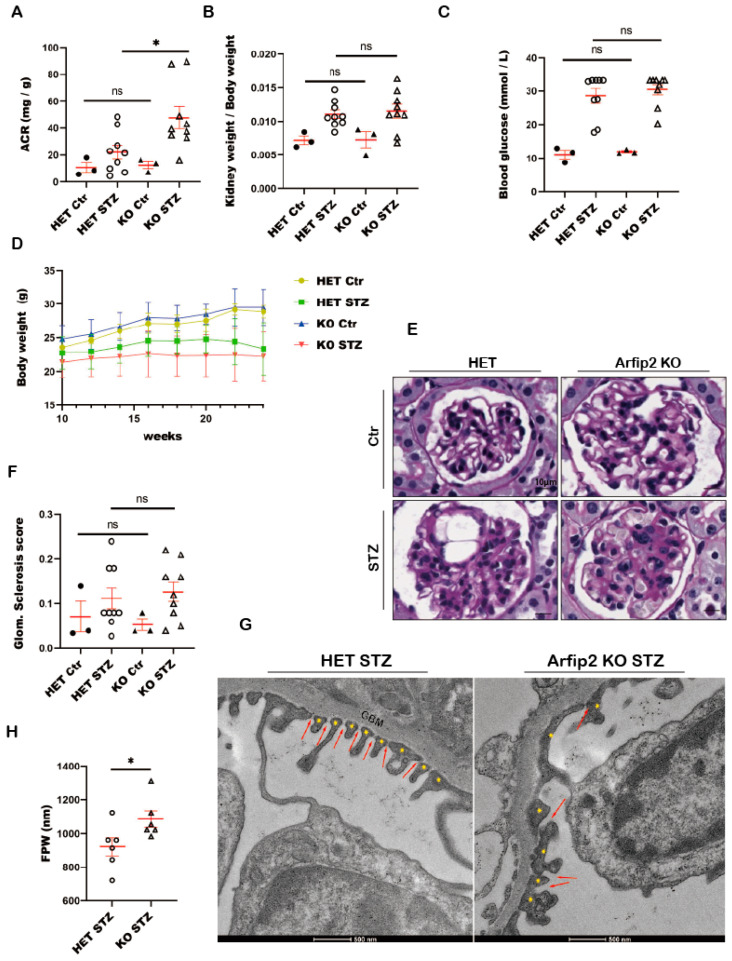
*Arfaptin-2* (*Arfip2*)-knockout mice develop albuminuria and subtle glomerular sclerosis in STZ-induced type I diabetic nephropathy. (**A**): The albumin/creatinine ratio (mg/g) in the urine of diabetic *Arfip2*-knockout mice increases significantly compared to diabetic heterzygous mice 16 weeks after diabetes induction (control group *n =* 3; STZ group *n =* 9). The Mann–Whitney test and two-tailed *t*-tests were used to analyze the control group and the STZ group, respectively. (**B**): The kidney weight/body weight ratio of diabetic *Arfip2*-knockout and diabetic heterzygous mice showed no significant difference. *t*-tests, two-tailed, were used for analyses. (**C**): Final blood glucose levels of diabetic *Arfip2*-knockout and diabetic heterzygous mice. The Mann–Whitney test was used in the STZ group. *t*-tests, two-tailed, were used for analysis in the control group. (**D**): The body weight of diabetic *Arfip2*-knockout and diabetic heterzygous mice after Streptozotozin injections. (**E**): Histological analyses (PAS stainings) of diabetic Arfip2-knockout and diabetic heterzygous mice. Scale bar 10 µm. (**F**): The quantification of glomerulosclerosis of diabetic *Arfip2*-knockout and diabetic heterozygous control mice. The Mann–Whitney test was used in the STZ group. *t*-tests, two-tailed, and used to analyze the control group. (**G**): The TEM of *Arfip2*-knockout and diabetic het control mice. Transmission electronmicroscopy (TEM) of diabetic *Arfip2*-knockout demonstrates the increased widening and misconfiguration (foot process (FP) effacement) of podocyte FPs (yellow asterisks). Slit diaphragm density (red arrows) is decreased, and slit diaphragms are partially translocated to proximal parts of FPs in diabetic *Arfip2*-knockout mice. The scale bar is 500 nm. (**H**): The mean width of the foot processes (FPWs) in diabetic *Arfip2*-knockout mice is higher than in diabetic heterzygous mice. Two-tailed *t*-tests were used for analyses. Heterozygote control mice (Het Ctr); *Arfip2* knockout mice (Arfip2 KO); heterozygote diabetic mice (Het STZ); diabetic *Arfip2* knockout mice (Arfip2 KO STZ). * *p* < 0.05.

To further investigate potential early podocyte damage based on the absence of Arfip2, we generated constitutive *Arfip2* knockout mice (Supplemental Figure S1) and confirmed successful *Arfip2* KO via in situ hybridization (Supplemental Figure S2). Constitutive *Arfip2*-KO mice did not show any developmental abnormalities or changes in kidney/body weight and exhibited no increase in proteinuria levels during a follow-up after 18 months. We investigated renal function using a model of type I diabetic nephropathy. One of the first indicators for podocyte damage and disturbance of the renal filter was the presence and accumulation of albumin in the excreted urine. The albumin/creatinine ratio (ACR) of diabetic *Arfip2* knockout mice (*n =* 9) was about 47.78 ± 8.27 mg/g and thereby significantly increased (*p* < 0.01) compared to heterozygous diabetic mice (*n =* 9) with an ACR of 22.16 + 5.12 mg/g (Figure 8A). Diabetic mice developed features of mild diabetic nephropathy (DN) (i.e., glomerular hypertrophy, hyperfiltration). However, there was no difference in kidney weight/body weight ratios (Figure 8B) and blood glucose levels (Figure 8C) between diabetic *Arfip2* knockout mice and diabetic heterozygous controls. Diabetic *Arfip2* knockout mice kept a lower body weight compared to diabetic heterozygous mice, but this difference was not significant (Figure 8D).

### 3.9. Arfip2 Knockout Mice Show Subtle Glomerular Sclerosis in Type I Diabetic Nephropathy

Due to the loss and retraction of their foot processes during the development of type I diabetic nephropathy, damaged podocytes were associated with a defective glomerular filtration barrier, and albumin was lost in the urine. Increased albuminuria in diabetic *Arfip2* deficient mice was accompanied by subtle histopathologic alterations, including podocyte and mesangial hyperplasia (Figure 8E). In semi-quantitative index analyses of glomerulosclerosis, we could not observe significant differences between diabetic *Arfip2* KO mice and diabetic heterozygous littermates (Figure 8F). However, further electron microscopic analyses revealed differences in podocyte homeostasis. Podocyte foot processes of diabetic Arfip2 KO mice showed a significant increase in widening and misconfiguration compared to diabetic littermate controls. Also, slit diaphragm density was decreased, and slit diaphragms were partially translocated to proximal parts of foot processes in diabetic KO mice (Figure 8G,H).

### 3.10. Autophagy Is Impaired in Constitutive Arfip2 Knockout Mice in Type I Diabetic Nephropathy

**Figure 9:**
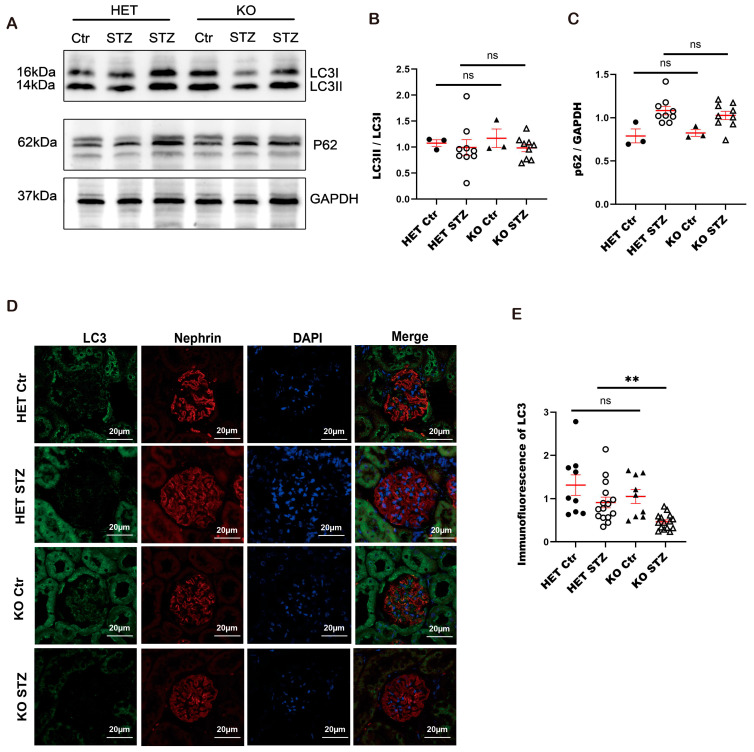
Autophagy is altered in STZ-induced type 1 diabetic nephropathy. (**A**): Western blot analyses showed microtubule-associated proteins 1A/1B light chain 3B I and II (LC3 I and II) and p62 protein levels in glomerular lysates of diabetic and non-diabetic *Arfaptin-2* (*Arfip2*)-knockout and heterozygous mice (Control group *n =* 3 STZ group *n =* 9). (**B**,**C**): Quantification of Western blot analyses. *t*-tests, two-tailed, and Mann–Whitney tests were used for analyses. (**D**): Confocal microscopic analyses and immunofluorescence staining for the LC3 of diabetic *Arfip2* knockout mice and diabetic heterozygous mice (*n =* 3). Scale bars 20 µm. (**E**): The quantification of immunofluorescence staining of LC3 of diabetic *Arfip2*-knockout mice and diabetic-heterozygous mice. *t*-tests, two-tailed, were used for analysis. Glyceraldehyde 3-phosphate dehydrogenase (GAPDH). Heterozygote control mice (Het Ctr); *Arfip2* knockout mice (Arfip2 KO); heterozygote diabetic mice (Het STZ); and diabetic *Arfip2* knockout mice (Arfip2 KO STZ). ** *p* < 0.01.

Our further investigation confirmed that autophagy is inhibited in diabetic *Arfip2*-deficient animals. We observed less LC3 accumulation in diabetic *Arfip2* KO mice and diabetic heterozygous controls in immunofluorescence analyses, though no difference in Western Blot (Figure 9A,B,D,E). However, there was no difference in the p62 levels in diabetic *Arfip2* KO mice and diabetic heterozygous controls. p62 was increased in both cases due to already-blocked autophagy under diabetic conditions (Figure 9A,C).

## 4. Discussion

The importance of the glomerular filtration barrier (GFB) lies in its role in maintaining the body’s internal environment, overall health, and homeostasis by selectively filtering the blood and forming urine. It consists of three main layers consisting of the glomerular endothelium, the glomerular basement membrane, and the podocytes with their interdigitating foot processes, which maintain high basal levels of autophagy [27] and work together to prevent the passage of larger molecules, such as proteins and blood cells into the urine while allowing the filtration of waste products and essential substances. If the glomerular filtration barrier is damaged or compromised, it can lead to various kidney disorders, such as glomerulonephritis or nephrotic syndrome, where proteinuria occurs.

Previous studies indicate that ARFIP2 might act as a component of ATG9A in starvation-induced autophagy [25]; however, its specific role remains elusive so far. In our study, we generated for the first time ARFIP2-deficient human podocytes and constitutive *Arfip2*-deficient mice to investigate the role of ARFIP2 in autophagy and mitophagy for podocyte homeostasis in vitro and in vivo. We show that ARFIP2 is differentially expressed in podocytes during autophagy (Figure 2). Importantly, the ATG9A protein is significantly decreased in ARFIP2-deficient podocytes (Figure 3C), which is consistent with the latest research [25].

ARFIP2-deficient podocytes exhibit increased levels of the lysosome-associated membrane glycoprotein LAMP2 (Figure 3E), p62/SQSTM1 [28], and LC3 (Figure 3D and Figure 5). These results are in line with our previous research on Class III PI 3-kinase (VPS34)-deficient podocytes, which is also a core regulator of autophagy, suggesting that autophagic flux was largely blocked in VPS34-deficient podocytes [29].

Under varying glucose conditions, ARFIP2-deficient podocytes show significantly more LCI in LC3II conversion in a 5.5 mM, 11 mM, and 30 mM glucose medium, defined as low, normal, and high glucose conditions and referring to a regular podocyte growth medium in vitro (Figure 4). p62 accumulates in low glucose conditions, indicating the impact of ARFIP2 deficiency in podocytes under deprived nutrition conditions (Figure 4). Several studies showed that high glucose can lead to impaired autophagy in podocytes and vice versa, suggesting that the activation of autophagy might protect podocytes against high glucose-induced insulin resistance and cell injury [30]. In our in vitro studies on ARFIP2-deficient podocytes, only low glucose conditions impaired autophagy significantly. We hypothesize that ARFIP2 is a regulator of starvation-induced autophagy. LC3II accumulation in isolated glomeruli and podocytes in diabetes has been seen in several studies [31].

Diabetic nephropathy (DN) is a serious and common microvascular complication of diabetes and is the leading cause of End Stage Renal Disease (ESRD) with the requirement of dialysis or transplantation in Western countries [32]. An increase in urinary albumin excretion is often the first sign of renal injury in diabetes. A key inducer of albuminuria is podocyte injury and/or loss. The pathological features of DN include the thickening of the glomerular basement membrane (GBM), mesangial expansion, nodular glomerulosclerosis, hypertrophy and the loss of podocytes, endothelial cell destruction [33] as well as the damage of the renal tubules and interstitium [34].

In a mouse model of DN induced by Streptozotozin (STZ) injections, we show that diabetic *Arfip2* KO mice have significantly increased ACR levels compared to diabetic heterozygous controls (Figure 8A–D), suggesting that Arfip2 deficiency deteriorates DN and further affects kidney function. Histopathological analyses did not show significant differences in glomerulosclerosis.

We assume that our analyses fell into an early stage of diabetic nephropathy, showing rather subtle pathological changes (i.e., the beginning of GBM thickening, mesangial expansion, and glomerulosclerosis). Electron microscopy revealed differences in the widening and misconfiguration of podocytes in diabetic *Arfip2* KO mice compared to diabetic littermate controls (Figure 8G), consistent with higher proteinuria in this group.

The development and progression of DN are triggered by multiple factors such as ROS production [35], endoplasmic reticulum (ER) stress [36], advanced glycation end-product formation, inflammation, and the activation of the renin-angiotensin system [37], all of which can trigger autophagy [38,39]. Here, we were able to detect attenuated autophagic activity in diabetic *Arfip2*-deficient mice. Arfip2-deficient mice show less LC3II (Figure 9), indicating that there is impaired autophagy flux in DN. Some studies suggest that autophagy can protect from diabetes-induced glomerulosclerosis and that, in DN vice versa, autophagy is disturbed [40,41]. This explains how impaired autophagy in *Arfip2*-deficient diabetic mice promotes glomerulosclerosis and more proteinuria. However, we did not observe more p62 accumulation in diabetic *Arfip2* deficient mice, although we expected p62 to be upregulated in diabetic mice, supporting the theory that autophagy is impaired. We hypothesize that this discrepancy between in vitro and in vivo results might be due to alternative pathways in vivo that clear accumulated p62, which is subject to further investigation in our laboratory.

Recent studies have highlighted the critical role of mitochondria in the maintenance of podocyte integrity as well as in the development and progression of podocytopathy, even though podocytes have lower numbers of mitochondria, i.e., proximal tubular epithelial cells [42,43]. Mitochondrial integrity is mediated by the balance between fusion and fission to maintain normal numbers and morphology. Mitochondrial fission in mammals is mediated by a large dynamin-like GTPase, known as Drp-1, which is recruited from the cytosol to form spirals around the mitochondria to constrict inner and outer membranes. Fusion is mediated by a single dynamin family member called Opa1 in mammals [44]. Here, we show that in ARFIP2 deficient podocytes, DRP-1 is upregulated and induces more fragmented mitochondria compared to wildtype controls (Figure 6D). We hypothesize that ARFIP2 deficiency impairs the fission of mitochondria. The impaired fission of mitochondria and PINK1/Parkin-induced mitophagy are in accordance with a recent study suggesting that fission is required for mitophagy [45]. The degradation and elimination of damaged mitochondria are mediated by mitophagy, a distinct form of autophagy.

A very recent study supports the idea that ARFIP2 participates in ATG9A trafficking during PINK1/Parkin-regulated mitophagy [26]. In addition, ATG4 regulates ATG9A trafficking independently of ATG8. We aimed to study the role of ARFIP2 in the mitophagy of podocytes [26]. Here, we observed an increase in PINK 1 and Parkin in ARFIP2-deficient podocytes (Figure 6E), suggesting that ARFIP2 deficiency promotes mitochondria injury and induces mitophagy in podocytes. We show that ARFIP2-deficient podocytes have decreased mitochondria lengths in comparison to wildtype controls (Figure 6A,B). Electron microscopic analyses further confirmed the fragmentation and decreased total number of mitochondria in ARFIP2-deficient podocytes (Figure 6C). Increasing evidence supports the idea that mitochondrial fission facilitates the induction of mitophagy [46,47]. Mitochondrial glycolysis and oxidative phosphorylation (OXPHOS) are the two major pathways to ensure energy supply in cells. Most cells can switch between both pathways for the purpose of coping with changing energy demands. Here, we show that ARFIP2 deficiency increases OXPHOS and ROS in podocytes (Figure 7A,J). Autophagy has earlier been linked to OXPHOS signaling. In our study, we observed enhanced oxidative respiration in ARFIP2-deficient podocytes, supporting the fact that cells could compensate for autophagy loss by upregulating their energy metabolism via OXPHOS.

## 5. Conclusions

In summary, we show that ARFIP2 is a novel regulator of autophagy and mitochondrial homeostasis in podocytes by facilitating ATG9A trafficking during PINK1/Parkin-regulated mitophagy. We hypothesize that Arfip2 deficiency mediates the dysregulation of mitophagy and autophagy in diabetic nephropathy, leading to cellular dysfunction, inflammation, fibrosis, apoptosis, senescence, and renal hypoxia. These mechanisms collectively contribute to the progressive deterioration of kidney function in individuals with diabetes, ultimately leading to diabetic nephropathy.

## Figures and Tables

**Table 1:** Primer sequences.

Primer Names	Gene Accession Numbers	Sequence
Arfip2_sonde1_ml	NM_029802.4	5′ CGC GGG ACG CGT AGT CCC TGC TCA CAG TGC TT 3′
Arfip2_sonde1_no	NM_029802.4	5′ CGC GGG GCG GCC GCC TGC AGT TGT CTT TGG ACC TG 3′
Arfip2_sonde2_ml	NM_029802.4	5′ CGC GGG ACG CGT GTC AGG ACC CAA CCT CAA TG 3′
Arfip2_sonde2_no	NM_029802.4	5′ CGC GGG GCG GCC GCC GTC TGT CCG GTA GGC ATC AT 3′
in situ mArfip2 KO_f	NM_029802.4	5′ ATC CAT CTC ACA GCA CGT CA 3′
in situ mArfip2 KO_r	NM_029802.4	5′ AAG CTC TGG GGA CTT CTG G 3′
mArfip2 KO_f	JN948356.1	5′ TGC AGG CAC ATA GAC ACA TAC AGG AA 3′
mArfip2 KO_r	JN948356.1	5′ GGA ACT TCA GCT TGA TGG CC 3′
mArfip2 HET_f	JN948356.1	5′ TGC AGG CAC ATA GAC ACA TAC AGG AA 3′
mArfip2 HET_r	JN948356.1	5′ TGT ACG AGA TAA GCG GGA ATA GAA GCA A 3′
mArfip2 flox_f	JN948356.1	5′ AGC TTC TCA TAC TTG TCC CGA T 3′
mArfip2 flox_r	JN948356.1	5′ AGC TTC TCA TAC TTG TCC CGA T 3′
gRNA_Arfip2	NM_012402.5	5′ GAG CGT GTC TTC CAT GGT CTT GG 3′
Crispr/cas9 hArfip2_f	NM_012402.5	5′ AAG ACC ATG GAA GAC ACG CTC 3′
Crispr/cas9 hArfip2_r	NM_012402.5	5′ CCC GAT GGG CCT GGA AAG 3′
5FRT_f		5′ AGG CGC ATA AAC GAT ACC ACG AT 3′
5FRT_r		5′ CCA CAA CGG GTT CTT CTG TT 3′
Arfip Ef (forward)	JN948356.1	5′ TGC AGG CAC ATA GAC ACA TAC AGG AA 3′
Arfip Er3 (reverse wildtype)	JN948356.1	5′ TGT ACG AGA TAA GCG GGA ATA GAA GCA A 3′
Arfip Kr (reverse mutant)	JN948356.1	5′ GGG CAA GAA CAT AAA GTG ACC CTC C 3′

**Table 2:** Antibodies.

Antibody/Fluorescent Compound	Dilution	Condition	Company
Rabbit anti-Arfapin 2 (40-2400)	1:1000	WB	Thermo Fisher Scientific, Germany
Rabbit anti-LC3B (#2775)	1:1000 1:100	WB IF	Cell Signaling Biotechnology Cambridge, UK
Guinea pig anti-nephrin (GP-N2)	1:100	IF	Progen Heidelberg, Germany
Guinea pig anti-p62 (GP62-C)	1:1000	WB	Progen Heidelberg, Germany
Rabbit anti-PINK 1 (BC100-494)	1:1000	WB	Novus Biologicals, USA
Rabbit anti-Parkin (14060-1-AP)	1:1000	WB	Proteintech, Manchester, UK
Mouse anti-TOM20 (F-10)	1:100	IF	Santa Cruz Biotech, Dallas, TX, USA
Rabbit anti-ATG9A (NB110-56893)	1:1000	WB	Novus Biologicals, USA
Rabbit anti-Lamp2 (ab37024)	1:1000	WB	Abcam, Berlin, Germany
Rabbit anti-GAPDH (14C10)	1:1000	WB	Cell Signaling Biotechnology, Cambridge, UK
Rabbit anti-Drp-1 (D6C7)	1:1000	WB	Cell Signaling Biotechnology, Cambridge, UK
Mouse anti-Opa-1(612607)	1:1000	WB	BD Transduction Laboratories™, Heidelberg, Germany
Alexa Fluor 488 donkey anti-mouse IgG	1:500	IF, pH9	Invitrogen, Karlsruhe, Germany
Alexa Fluor 488 donkey anti-rabbit IgG	1:500	IF, pH9	Invitrogen, Karlsruhe, Germany
Alexa Fluor 555 donkey anti-guinea pig IgG	1:500	IF, pH9	Invitrogen, Karlsruhe, Germany
Hoechst	1:5000	IF, pH9	Molecular Probes, Eugene, OR, USA
Goat anti-rabbit IgG-HRP (#7074S)	1:3000	WB	Cell Signaling Biotechnology Cambridge, UK
Goat anti-mouse IgG-HRP (#P0447)	1:10,000	WB	Dako, Glostrup, Denmark
Goat anti-guinea pig (A0564)	1:8000	WB	Dako, Glostrup, Denmark

## Data Availability

The data presented in this study are available on request from the corresponding author.

## Supplementary Materials

The following supporting information can be downloaded at: https://www.mdpi.com/article/10.3390/antiox13010081/s1, Figure S1: Creation of a constitutive *Arfip2* knockout mouse model using the Cre-lox and FLP-FRT systems; Figure S2: Confirmation of successful *Arfip2* knockout in mice.
