# Supplementary material for: ADP-Ribosylation Factor-Interacting Protein 2 Acts as a Novel Regulator of Mitophagy and Autophagy in Podocytes in Diabetic Nephropathy
Source: Antioxidants (Basel). 2024 Jan 8;13(1):81. doi: 10.3390/antiox13010081 (PMC10812550; doi:10.3390/antiox13010081)

Supplementary Data

Supplementary Figure S1. Creation of a constitutive *Arfip2* knockout mouse model using the Cre-lox and FLP-FRT system

C57BL/6N-Arfip2tm1e(EUCOMM)Wtsi/Ieg mice carry a L1L2_Bact_P cassette at position 77267363 of Chromosome 5 upstream of the critical exons 4 and 5. The L1L2_Bact_P cassette is composed of an FRT site followed by lacZ sequence and a loxP site. This first loxP site is followed by a neomycin resistance gene under the control of the human beta-actin promoter, SV40 polyA, a second FRT site and a second loxP site. A third loxP site is inserted downstream of the targeted exon(s) at position 77269113. The critical exons 4 and 5 are thus flanked by loxP sites. A: C57BL/6NArfip2$^{tm1e(EUCOMM)Wtsi}$/Ieg mice were crossed to B6.129S4-Gt(ROSA)26Sor $^{tm2(FLP*)Sor}$/Jmice expressing the flp recombinase to create a conditional ready (floxed) allele. B: Subsequent crossings of the resulting mice with Sox2-cre (STOCK Edil3$^{Tg(Sox2-cre)1Amc}$/J) mice (a kind gift of Prof. S. Arnold, Pharmacology Department, University of Freiburg) result in a constitutive Arfip2-knockout mouse. Briefly, Sox2 (SRY-box containing gene 2) functions as a transcription factor that is essential for maintaining self-renewal or pluripotency of undifferentiated embryonic stem cells (1). Under control of the Sox2 promotor cre is expressed very early during development leading to a deletion of the floxed region in all cells. C: To confirm successful genetic knockout of Arfip2 in these mice, we crossed Arfip2$^{tm1e(EUCOMM)Wtsi}$ mice to Six2-TGCtg Tg(Six2-EGFP/cre)^{1Amc}/J animals (purchased from Jackson Laboratory (Bar Harbor,

ME, USA)). If cre expression occurs without flp expression, a reporter knockout mouse will be created.

Supplementary Figure S2. Confirmation of successful *Arfip2* knockout in mice.

A: To confirm successful genetic knockout of *Arfip2* in these mice, we performed *in situ* hybridization of E13.5 old mice. Scale bar 400µm. B - E: Renal cortex and medulla from wildtype mice. Scale bar 100µm. F: Confirmation of successful *Arfip2* KO was performed by mRNA in situ hybridization. G - J: Renal cortex and medulla from *Arfip2* knockout mice. Scale bar 100µm. K: Genotyping results for heterozygotes and *Arfip2* knockout mice. The band for heterozygotes mice is located at 398bp, the band for *Arfip2* knockout mice is located at 933bp. RNase-free Water was used for negative control.

Reference

1. Feng R, Wen J. Overview of the roles of Sox2 in stem cell and development. Biol Chem. 2015;396(8):883-91.

Supplemental Figure S1

A

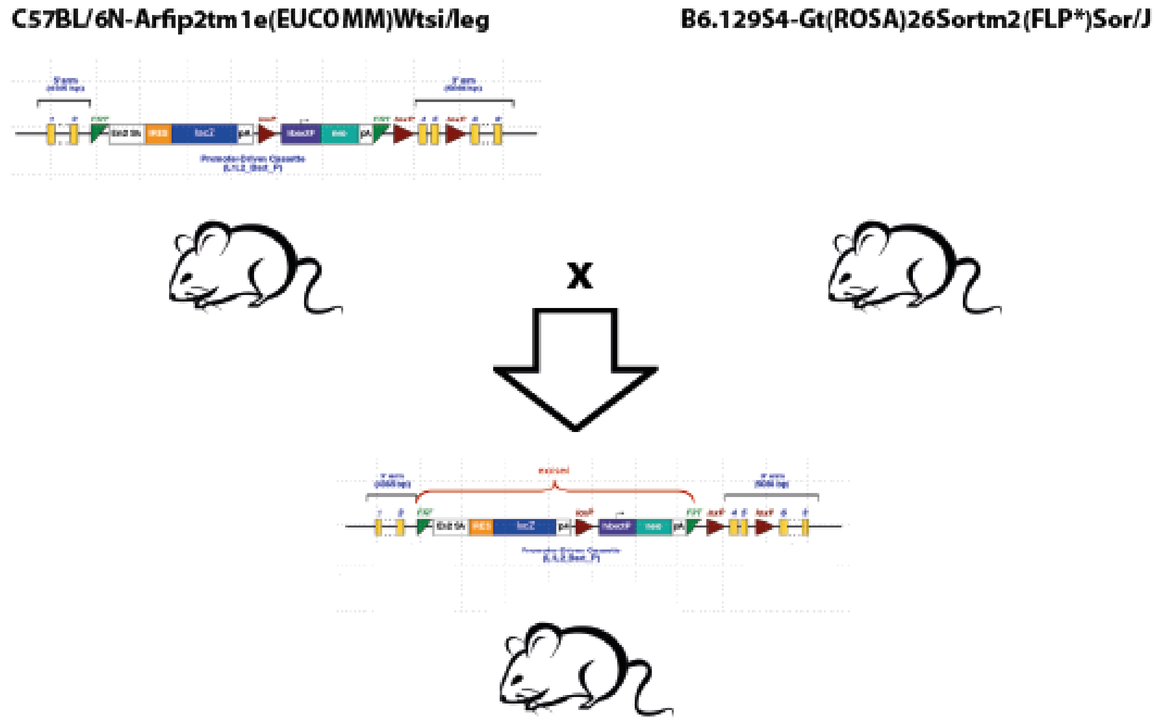

B

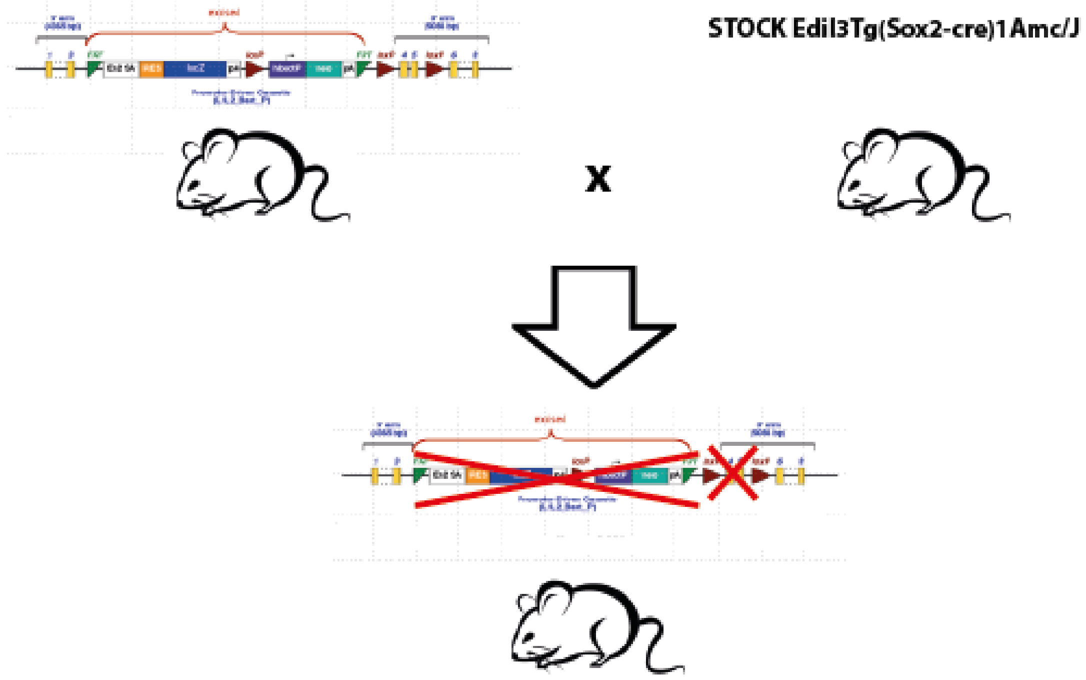

Arfip2$^{flox/flox}$ mouse

Supplemental Figure S2

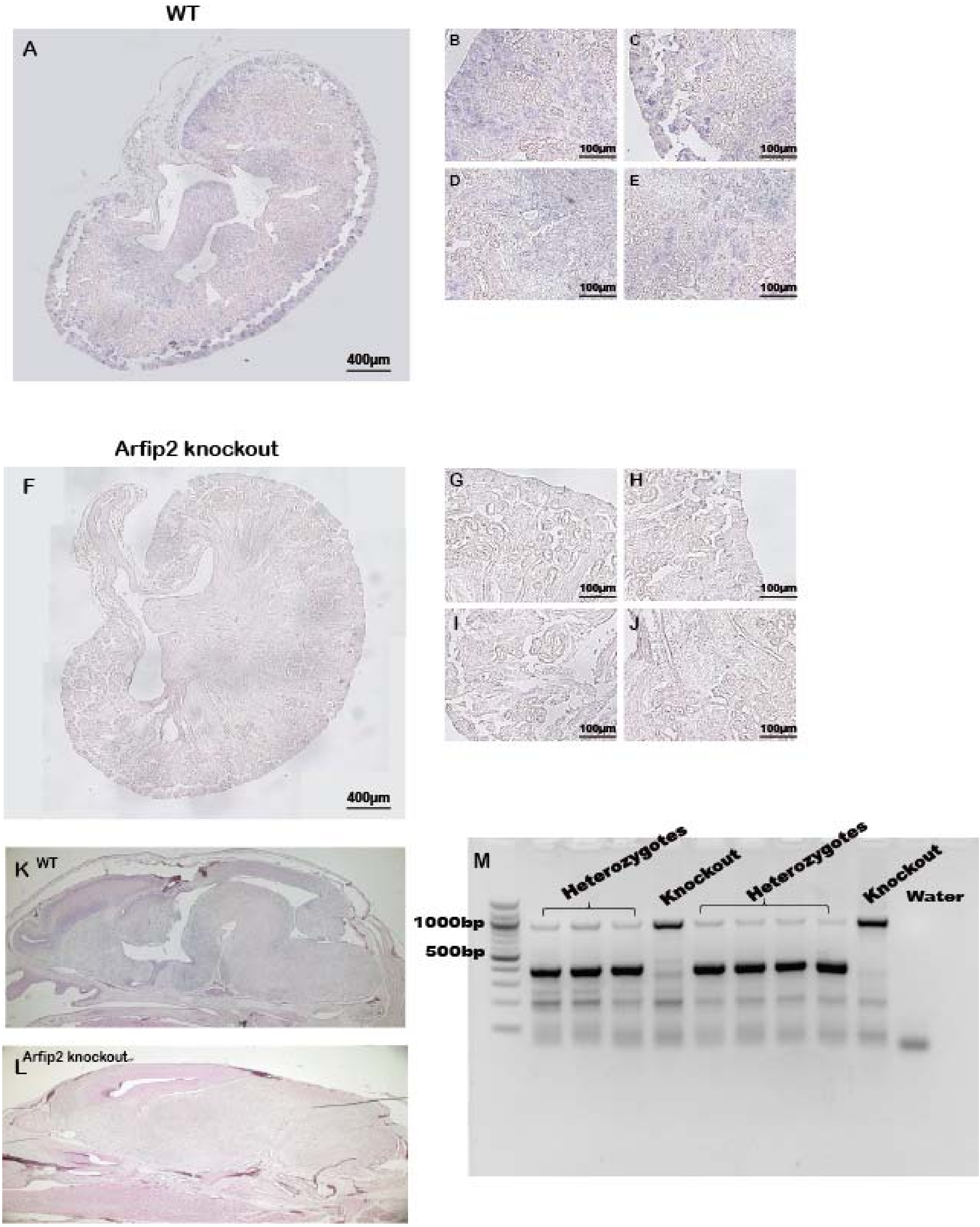

Supplement: Supplementary file 1 [file antioxidants-13-00081-s001.zip › antioxidants-2775266-supplementary.pdf]
